# Exploring the Link between ADHD and Obesity: A Focus on Temperament

**DOI:** 10.3390/brainsci12121631

**Published:** 2022-11-29

**Authors:** Maria Cristina Porfirio, Roberta Campanile, Gabriele Masi, Diane Purper-Ouakil, Silvia Giovinazzo, Alessandra Ascenzi, Alfonso Troisi, Luigi Mazzone

**Affiliations:** 1 Child Neurology and Psychiatry Unit, Tor Vergata Hospital, Fondazione PTV, Oxford Street 81, 00133 Rome, Italy; 2 Systems Medicine Department, University of Rome Tor Vergata, Montpellier Street 1, 00133 Rome, Italy; 3 IRCCS Stella Maris Foundation, Department of Child and Adolescent Psychiatry and Psychopharmacology, 56128 Pisa, Italy; 4 Unit of Child and Adolescent Psychiatry (MPEA1), CHU Montpellier-Saint Eloi Hospital, School of Medicine, 34090 Montpellier, France; 5 International Medical School, University of Rome Tor Vergata, 00133 Rome, Italy

**Keywords:** attention deficit hyperactivity disorder, obesity, temperament, dimensional assessment, harm avoidance, internalizing symptoms

## Abstract

Multiple studies support the relationship between ADHD and overweight/obesity in youth. Different mechanisms may be involved, such as temperamental and psychopathological factors. The aim of this study was to test the hypothesis that specific temperamental and psychopathological dimensions could mediate the relationship between ADHD and obesity. The sample included 100 children and adolescents (78 males and 22 females; age range 6 to 18 years; mean age 9.90 ± 2.5 years). The assessment procedure included Conners’ Parent Rating Scale—Long (CPRS-R:L) as the inclusion criterion for ADHD diagnosis, the Child Behavior Checklist (CBCL), a dimensional measure for psychopathology, and the Junior Temperament and Character Inventory, which describes four temperamental dimensions: novelty seeking (NS), harm avoidance (HA), reward dependence (RD), and persistence (P). While in the whole ADHD sample, the highest scores were found in NS and the lowest in P, ADHD with overweight/obesity, compared to ADHD with normal weight, showed higher HA and RD, lower NS, and higher CBCL Internalizing scores. These findings suggest that ADHD youth with overweight/obesity present specific temperamental and psychopathological features compared to those without overweight/obesity. If confirmed in larger samples, using a control group without ADHD, these temperamental and psychological features may be helpful for an earlier recognition of ADHD patients at higher risk for obesity, and may represent possible targets for temperament-based preventive interventions and tailored treatment programs. These features should be included in the routine assessment of children and adolescents with ADHD and/or are overweight/obese.

## 1. Introduction

Attention-deficit/hyperactivity disorder (ADHD) is characterized by age-inappropriate symptoms of inattention, hyperactivity/impulsivity, or both, which negatively impact academic, social, and domestic relationships [1]. ADHD is one of the most common behavioral disorders in children, with an estimated community prevalence between 2% and 7%, with an average of around 5% and a male prevalence [2].

The multifaceted nature of ADHD is expressed by the heterogeneity of its clinical presentations, namely the co-occurrence of emotional dysregulation in at least 40% of the patients, with implications not only in clinical expression but also in comorbidities (both psychiatric and non-psychiatric) and in response to treatments [3]. One of the aspects of this heterogeneity of ADHD is the comorbidity with other psychiatric disorders that contribute to the functional impairment of the patient, such as oppositional defiant disorder, conduct disorder, anxiety disorders, mood disorders, sleep disorders, and speech and learning disorders [4,5,6]. Beyond psychiatric comorbidities, common medical and metabolic conditions, namely overweight/obese [7,8,9], have been more frequently reported in ADHD youth and adults compared with the general population.

Overweight/obesity are major health problems, a sort of global epidemic, contributing significantly to both morbidity and mortality. According to the Centers for Disease Control and Prevention’s (CDC), a body mass index (BMI) at or above the 84th percentile is considered overweight, instead of a BMI at or above the 95th percentile.

Pathophysiological, environmental, and psychological mechanisms are usually mutually involved in pediatric obesity, and sometimes psychiatric disorders are co-occurring [10]. Multiple studies support the relationship in youth between ADHD and body weight dysregulation [11,12] higher than average body mass index (BMI), a higher percentage of body fat and abdominal circumference, and abnormal eating behaviors [13,14,15,16]. Of note, non-medicated patients with ADHD present with a higher risk of obesity [8,17].

Consistently, ADHD was found to be significantly more prevalent in clinically ascertained obese children [18,19,20], adolescents [20,21,22,23,24], and adults [25,26,27] when compared to those who are not obese. In addition, both obese subjects and patients with eating disorders are more likely to present attention problems [28,29].

The relationship between ADHD and overweight can be primarily interpreted in the light of impulsivity and emotional dysregulation [30], with low tolerance for delay and frustration. Furthermore, deficits in the reward system have been reported in patients with ADHD, which may also account for the ADHD-obesity connection [31,32]. However, other mechanisms may be involved, such as common neurobiological abnormalities (i.e., abnormalities in the brain dopamine systems) [12].

Temperament and personality traits frequently associated with ADHD like ‘neuroticism’ or ‘early negativity’, ‘impulsivity’, and ‘sensitivity to reward’ are possible risk factors for obesity, while ‘conscientiousness’ (regulation of internal urges and self-discipline, which may favor a greater control over impulsive and reward-oriented behavior) and ‘self-control’, which are poorly expressed in ADHD patients, have been shown to have a protective function in relation to weight gain [33]. An assessment of these temperamental features may be of interest in exploring the link between ADHD and obesity and in helping to timely recognize at-risk patients.

A comprehensive model to assess temperamental features of personality is Cloninger’s biopsychosocial theory of personality, based on the assumption that personality involves four temperament dimensions, novelty-seeking (NS); harm-avoidance (HA); reward dependence (RD), and persistence (P), and three character dimensions, self-directedness (SD), cooperativeness (C), and self-transcendence (ST) [34]. The four temperament dimensions measure individual differences in basic emotional drives, such as pessimistic and anxious versus optimistic and risk-taking (harm avoidance), impulsive and irritable versus rigid and stoical (novelty seeking), sociable and warm versus aloof and cold (reward dependence), and persevering and ambitious versus easily discouraged and lazy (persistence). This model can be used to characterize temperamental features of ADHD patients as a group [35], but also to disentangle specific subgroups of ADHD patients at higher risk for specific comorbidities, including obesity. Specific areas of the prefrontal cortex (including the dorsolateral prefrontal, anterior cingulate, and orbitofrontal cortices) and limbic structures (including the amygdala, hippocampus, and nucleus accumbens) are “key regions” associated with three fundamental dimensions of temperament: “Negative Affect”, “Positive Affect”, and “Constraint” [36].

A “typical ADHD temperament profile” in children and adolescents with ADHD is characterized by high NS in combination with low RD, SD, C, and P [37,38,39,40]. The data about HA are more inconsistent, as some studies report high scores of HA while others have shown no significant differences between ADHD patients and healthy controls [41], although different patterns of comorbidity (i.e., internalizing vs. externalizing disorders) may account for these discrepancies.

NS represents the tendency to respond with excitement to novel stimuli or cues for potential rewards, which leads to frequent exploratory activities in pursuing potential rewards as well as an active avoidance of monotony [40]. Some studies [42] point to the role of temperament (NS) in mediating the relationship between ADHD and personality disorders, such as borderline personality disorders (BPD), addiction disorders such as alcohol and nicotine, substance abuse, gambling, and obesity.

HA, instead, is a heritable tendency to respond intensely to signals of aversive stimuli, thereby learning to inhibit behavior to avoid punishment, novelty, and frustrative non-reward. HA is related to personality-associated traits of negative affect such as anxiety, depression, fear, shyness, constant concern, neuroticism, and negative affectivity [43]. Subjects with high levels of NA have a negative mood and are impulsive, which may influence the sudden and uncontrolled increase in appetite, snack food meals, and weight, and also play a key role in addictive-like eating behavior (i.e., binge eating, food addiction, and loss of control overeating) [12].

Consistent with these reports, according to Cloninger’s model of personality, obese individuals, compared with normal-weight subjects, should present a distinctive temperament profile, characterized by low SD and C and high NS and HA. In obese individuals with associated binge eating disorder, this profile is even more pronounced [44,45]. These temperamental dimensions may be related to psychopathological dimensions. A dimensional approach to psychopathology, based on the continuity between disorders and normality, can give more informative descriptions for understanding risk and predicting outcome than traditional diagnostic categories, which are often heterogeneous and overinclusive [46]. Multiple studies that shaped the Achenbach System of Empirically Based Assessment (ASEBA) [47], supported the important distinction between broad internalizing and externalizing spectra and their implication, i.e., in the prediction of early substance use [48]. The Child Behavior Checklist (CBCL), the gold standard for the study of dimensional psychopathology in children and adolescents, has been used in obese and overweight children [49]. Overweight children, compared to non-overweight controls, presented higher scores in social problems, defiant problems, and total problems, while children at risk of being overweight showed higher scores only in social problems compared to not overweight children.

The aim of our study was to explore the role of both temperament and psychopathological dimensions in youth with ADHD. Our hypothesis is that specific temperaments, such as RD, and psychopathological dimensions, such as internalizing symptoms, may mediate the relationship between ADHD and obesity. If these features differentiate ADHD patients with and without overweight/obesity, they may represent possible targets for the diagnostic assessment and the treatment strategies.

## 2. Materials and Methods

### 2.1. Participants

Our sample included all the 100 drug-naïve children and adolescents with ADHD referred and consecutively diagnosed at the Child and Adolescent Psychiatric Unit of Tor Vergata Hospital from 2018 to 2020. The sample consisted of 78 males and 22 females, ranging in age from 6 to 18 years, with a mean age of 9.9 ± 2.5 years, 67 with combined presentation, 29 with inattentive presentation, and 4 with hyperactive/impulsive presentation. Socio-economic status (SES) was assessed with the Barratt Simplified Measure of Social Status (BSMSS). 

The diagnosis was based on the DSM-5 criteria and the Conners’ Parent Rating Scales and Teacher (Italian version) as part of a comprehensive clinical assessment by a trained multidisciplinary team. All the patients taking any meditations or with metabolic or endocrinological diseases were excluded, as were patients with intellectual disability, autism spectrum disorder, schizophrenic spectrum disorder, bipolar disorder, personality disorder, and substance use disorder.

All the participants received a comprehensive assessment, including also the Kiddie Schedule for Affective Disorders and Schizophrenia for school-age children (K-SADS-PL) [50] for categorical psychiatric diagnoses, the Wechsler Intelligence Scale for Children (WISC-IV) [51] for intellectual functioning, and the Conners’ Parent Rating Scales and Teacher (Italian version) for the diagnosis of ADHD. For the purposes of our study, weight and growth, dimensional psychopathology, and temperament/character were specifically explored, and related measures will be presented in the next section. 

The methodology of the study was clearly presented to patients and parents, and a written informed consent to participate was obtained by the parents of all the participants.

The study protocol was approved and registered (R.S. #216.20) by the local institutional review board (Rome Tor Vergata University-Hospital Ethical Review Committee).

### 2.2. Measures

#### 2.2.1. Growth Measurement and Assessment

Children’s height and weight (without shoes, hats, and heavy outer clothing such as jackets or sweaters) were measured during their first visit. The indicators of BMI (weight in kilograms divided by height in meters squared) percentiles and BMI z-score were used to determine the individuals’ growth. BMI percentiles and z-scores were considered based on the World Health Organization (WHO) age/gender references available on the WHO website(https://www.who.int/news/item/27-04-2006-world-health-organization-releases-new-child-growth-standards/). The WHO defines “normal weight” as ranging between the 3rd to 84th percentile, “overweight” as ranging between the 85th to 96th percentile, and “obesity” as equal to or greater than the 97th percentile.

#### 2.2.2. ADHD Symptoms

The Conners’ Parent Rating Scale—Long (CPRS-L) [52], Italian version [53], was used to select the sample and assess children’s current ADHD symptoms. The long parent version used for children aged 6–18 years includes 80 items, and each item is rated on a four-point scale concerning the frequency of occurrence (never, occasionally, often, very often) to establish the severity of ADHD symptoms. The measure yields t-scores on ten different scales: Oppositional, Cognitive Problems/Inattention, Hyperactivity, Anxiety-Shy, Perfectionism, Social Problems, Problems psychosomatic Conner’s Global Index, Index and Symptoms of ADHD DSM-5. According to the standardization, T scores greater than or equal to 60 are considered pathological.

#### 2.2.3. Dimensional Psychopathology

The Child Behavior Checklist 6–18 (CBCL) [47], Italian version [54], a parent-report version, was administered to assess children’s behavioral and emotional problems. It is used for subjects aged 6 to 18 years and assesses internalizing and externalizing behaviors with eight syndrome scales, including social withdrawal, somatic complaints, anxiety/depression, social problems, thought problems, attention problems, aggressive behavior, and rule-breaking behaviors. The raw scores obtained are converted into T-scores, and according to the standardization, a T-score ≥ 63 is considered clinically significant in the three scales internalizing, externalizing, and total, while in the syndromic scales this cut-off is equal to ≥70. Analysis of the psychometric properties of the instrument showed good internal consistency and reliability [55].

#### 2.2.4. Temperament and Character

The Junior Temperament and Character Inventory (JTCI) [56], a 107-item questionnaire, with both parent- and child-versions in its Italian version [57], was used to assess the biogenetic temperament and character of children 6–16 years old. In the present study, only the parent’s rating version was completed by both mother and father for each participant, reporting their perceptions of their children’s behaviors, opinions, and feelings [58]. The items measure the seven dimensions of the Cloninger psychobiological model of personality [34]. The four temperament factors, explored by different items on the JTCI, are novelty seeking (NS), harm avoidance (HA), reward dependence (RD), and persistence (P). The three character factors are self-directedness (SD), cooperativeness (CO), and self-transcendence (S).

#### 2.2.5. Statistical Analysis

Statistical analysis included one-way analysis of variance (ANOVA) and covariance (ANCOVA). To minimize the likelihood of a type-I error, univariate ANOVAs were preceded by an overall multivariate analysis of variance (MANOVA) using all the JTCI or CBLC scales as dependent variables. The significance of the MANOVA effect was assessed employing the F-test in association with Wilk’s lambda. Hierarchical regression analysis was used to identify the significant predictors of BMI in the entire sample. Collinearity diagnostics based on the eigenvalues of the scaled and uncentered cross-products matrix, variation inflation factors (VIF) and tolerances for individual variables were used to exclude multicollinearity among the independent variables. The software we used was SPSS version 25.0. The algorithms used by the software SPSS 25.0 for computing the multivariate analysis are proprietary algorithms reported in the software manual.

## 3. Results

### 3.1. Descriptive Preliminary Analysis 

All the variables of the study and their descriptive statistics are shown in Table 1.

Preliminary analyses did not show any significant association between demographic variables and children’s severity of ADHD symptoms or BMI z-scores.

Based on WHO-age norms 35, children whose BMI was above the ≥85th percentile were classified as overweight/obese, while children between the 3rd and 84th percentile were classified as normal weight.

The prevalence of overweight/obesity was 71/100 (46 were obese, which is beyond the 96th centile). None in the sample had a BMI < 3rd percentile.

Regarding the temperament profile in the whole ADHD sample, according to the JTCI, the highest scores were found in NS and the lowest in P and SD. Specifically, the NS trait was present in 86% of the sample, and the P trait in 11%.

### 3.2. Hierarchical Regression Analysis

The role of temperament in influencing the BMI of ADHD children was assessed with a hierarchical stepwise regression analysis using the three scales of the JTCI, formerly included by Cloninger in the Tridimensional Personality Questionnaire (TPQ) and more closely related to ADHD, that is, novelty seeking, harm avoidance, and reward dependence, as predictors. Persistence was excluded to reduce predictors’ numbers and increase the validity of the multivariate analysis, considering the sample size.

The variables entered in the first block were age and gender. The three scales of the JTCI comprised the second block. The multivariate model was significant (F = 3.21, df = 3, 96, *p* = 0.03, R squared = 0.09) and the HA scale of the JTCI emerged as a significant predictor of the BMI (Beta = 0.25, *p* = 0.01). See Table 2.

The relationship between BMI and psychopathological dimensions was assessed using a hierarchical stepwise regression analysis with the three broad-band scales of the CBCL, internalizing, externalizing, and total problems, as predictors. The variables entered in the first block were age and gender. The three scales of the CBCL entered the second block. The multivariate model was significant (F = 3.98, df = 3, 96, *p* = 0.01, R squared = 0.11) and the internalizing scale of the CBCL emerged as a significant predictor of the BMI (Beta = 0.29, *p* < 0.01). See Table 3.

To make our results comparable with those of previous studies, we repeated the statistical analysis using a categorical definition of overweight/obesity (coded as 1 = non-overweight, 2 = overweight) based on the WHO growth reference data for children and adolescents.

The role of temperament was assessed by conducting a MANOVA with the three scales of the JTCI (NS, HA, and RD) as dependent variables. The omnibus test was significant (Wilks’ Lambda = 0.91, F = 2.98, df = 3, 96, *p* = 0.03). Compared to non-overweight subjects, overweight/obesity subjects scored higher on the RD (F = 4.46, *p* = 0.04) and lower on the NS scale (F = 4.88, *p* = 0.03).

The role of behavioral problems was assessed by conducting a MANOVA with the three broad-band scales of the CBCL, internalizing, externalizing, and total problems, as dependent variables. The omnibus test fell short of statistical significance (Wilks’ Lambda = 0.94, F = 2.10, df = 3, 96, *p* = 0.11).

## 4. Discussion

Although the relationship between ADHD and overweight/obesity has been documented, the mechanisms underlying this connection are still being discussed. The aim of our study was to explore the role of temperamental and psychopathological dimensions as possible factors affecting this relationship. A better understanding of these mechanisms may lead to earlier recognition of at-risk subjects and more timely and personalized treatments. Our results confirm previous research, supporting the notion that ADHD and overweight are highly comorbid, as, according to our findings, up to 71% of the patients in our ADHD sample were overweight or obese [7,8,9].

### 4.1. This Work Summary

As expected, the most frequent temperamental feature in the ADHD sample as a whole was novelty seeking (NS), while the least represented were persistence (P) and self-directedness [36,37,38]. Regarding the temperamental dimension more closely related to the risk of being overweight/obese, harm avoidance (HA) was the best predictor of a higher BMI. Consistently, internalizing disorders were the psychopathological dimension that mostly predicted a higher BMI. Compared to non-overweight subjects, overweight/obesity subjects as a category scored higher on the reward dependence (RD) scale and lower on the NS scale.

### 4.2. Contributions

Subjects with high RD tend to strongly respond to signals of reward, and when this feature is particularly high, it may increase the development of psychopathological traits like the risk of addictive behavior or overeating, as in our sample. These results are consistent with the notion that dysregulated brain reward pathways may be contributing not only to drug addiction but also to increased intake of palatable foods and ultimately obesity [59]. Obesity may be associated with altered reward functions, although it is not clear whether these changes are the cause or consequence of obesity. In other words, RD may be a precursor of abnormal eating behaviors, and a vulnerability marker, or the overweight condition may determine both the temperamental and psychopathological dimensions. Both conditions may be true, and each may characterize a different type of patient.

In comparison to the personality variables that influence later substance use, such as high NS and RD, low HA and negative affectivity, our patients with obesity have low NS and high HA, as well as prevalent internalizing disorders. In other words, both ADHD subjects with obesity and those with substance abuse may present high RD, but while subjects prone to substance abuse may present high NS and low HA (impulsive, quick-tempered, intolerant to frustration and rules, low dopaminergic activity, and typical ADHD features), patients with obesity are characterized by low NS and high HA (excessive worrying, pessimism, shyness, being fearful, doubtful, and easily fatigued), with neurotic introversion more closely related to the typical features of binge- and over-eating [44,45]. This is consistent with previous findings linking depression and negative affects to ADHD and the development of childhood obesity [12].

### 4.3. Limitations and Strengths

Our study should be considered in the light of several limitations, mainly the small sample size, the lack of differentiation of ADHD’s subtypes and their different temperament profiles, and the lack of a control group of patients without ADHD, to ascertain whether the psychopathological and temperamental features may be related to the ADHD diagnosis. Given the small sample size, gender differences were not considered, as the girls were under-represented, consistent with the ADHD gender ratio. Furthermore, in our study, we focused on the three broad-band scales of the CBCL, without a closer consideration of specific categorical diagnoses. The main strength of the study is that it included an unselected sample of drug-naïve ADHD youth in routine clinical conditions; thus, the results may more closely reflect the real-life context.

### 4.4. Future Work

Our preliminary study suggests that ADHD children and adolescents, with or without obesity significantly differ according to their temperamental and psychopathological profiles. Further research with larger samples, a longitudinal design, and different measures may support these preliminary results. In clinical settings, diagnostic and treatment implications may be drawn more consistently. A comparison between overweight/obese children and adolescents with and without ADHD may further support the specificity of our findings. Among the new measures, a body shape index (ABSI) may replace the old BMI as an index of obesity, which is becoming outdated as a result of different medical fields [60,61,62].

## 5. Conclusions

ADHD subjects with lower BMI have a predominant NS temperament and externalizing symptoms, whereas ADHD subjects with higher BMI have a high RD and HA temperament and associated internalizing symptoms. This profile, markedly different from that of patients with other addictive behaviors, such as substance abuse (except for the shared RD), may represent a possible marker of vulnerability in ADHD patients. If confirmed in larger, prospective studies, this finding may underline the need to include a dimensional approach to temperament and psychopathology in a systematic assessment of children with ADHD, to improve our ability to identify more at-risk subjects, and to tailor interventions to temperament-based prevention programs (such as mindfulness-based programs) [63], according to the presence of high HA but not high NS. Similarly, in youth with ADHD and obesity that are co-occurring, an investigation of both the temperamental features of HA and internalizing symptoms may address more specific treatment strategies compared to patients with ADHD without obesity.

## Figures and Tables

**Table 1:** Descriptive statistics for all the variables included in the study.

	Mean	SD	Minimum	Maximum
ADHD severity				
CPRS ADHD index	72.12	13.30	38	100
CPRS Inattent. Index	70.73	12.32	38	99
Temperament				
JTCI-NS	11.22	3.21	1	17
JTCI-HA	8.32	4.49	1	22
JTCI-RD	5.74	2.07	0	9

Legend: CPRS: Conners’ Parent Rating Scales; JTCI: Junior Temperament and Character Inventory; NS: Novelty Seeking; HA: Harm Avoidance; RD: Reward Dependence.

**Table 2:** Results of a hierarchical regression analysis with Body Mass Index (BMI) as the dependent variable and the Junior Temperament and Character Inventory (JTCI) scales as predictors (N = 100).

		Β	t	*p*
Step 1	Gender	−0.10	−1.03	0.303
	Age	−0.15	−1.51	0.135
	Model	R^2^ = 0.03	F = 1.47	0.235
Step 2	Gender	−0.11	−1.16	0.248
	Age	−0.15	−1.49	0.140
	HA	−0.25	−1.80	0.012
	NS	−0.10	−0.95	0.344
	RD	0.05	0.50	0.620
	Model	ΔR^2^ = 0.06	ΔF = 6.52	0.012
		R^2^ = 0.09	F = 3.21	0.027

Legend: HA: JTCI Harm Avoidance; NS: JTCI Novelty Seeking; RD: JTCI Reward Dependence; B, unstandardized regression coefficient; t: t-value; *p*: *p*-value.

**Table 3:** Results of a hierarchical regression analysis with BMI as the dependent variable and the Child Behavior Checklist (CBCL) broad band scales as predictors (N = 100).

		Β	t	*p*
Step 1	Gender	−0.10	−1.03	0.303
	Age	−0.15	−1.51	0.135
	Model	R^2^ = 0.03	F = 1.47	0.235
Step 2	Gender	−0.05	−0.46	0.644
	Age	−0.15	−1.51	0.134
	INT	0.29	2.96	0.004
	EXT	−0.04	−0.30	0.763
	TOT	−0.03	−0.23	0.815
	Model	ΔR^2^ = 0.08	ΔF = 8.76	0.004
		R^2^ = 0.11	F = 3.98	0.010

Legend: INT: CBCL Internalizing problems; EXT: CBCL Externalizing problems; TOT: CBCL Total problems; B, unstandardized regression coefficient; t: t-value; *p*: *p*-value.

## Data Availability

The data presented in this study are contained within the article.

## Abbreviations

Attention Deficit Hyperactivity Disorder (ADHD); Conners’ Parent Rating Scale—Long (CPRS-R:L); Child Behavior Checklist (CBCL); Junior Temperament and Character Inventory; novelty seeking (NS); harm avoidance (HA); reward dependence (RD); persistence (P); Self-Directedness (SD), Cooperativeness (C), and Self-Transcendence (ST); body mass index (BMI); deficient emotional self-regulation (DESR); Borderline Personality Disorders (BPD); Socio-economic status (SES); Barratt Simplified Measure of Social Status (BSMSS); Schedule for Affective Disorders and Schizophrenia for school-age children (K-SADS-PL); Wechsler Intelligence Scale for Children (Fourth Edition) (WISC-IV).
